# Targeting RIG-I or STING promotes epithelial regeneration

**DOI:** 10.18632/oncotarget.22994

**Published:** 2017-12-06

**Authors:** Julius C. Fischer, Hendrik Poeck

**Affiliations:** Department of Radiation Oncology, Klinikum rechts der Isar, Technical University of Munich, Munich, Germany; Klinik und Poliklinik für Innere Medizin 3, Hämatologie und Onkologie, Klinikum rechts der Isar, Technical University of Munich, Munich, Germany

**Keywords:** RIG-I, STING, type I interferon, graft-versus-host disease, epithelial regeneration

Targeting pathways that enhance tissue repair such as regeneration of intestinal epithelial cells (IEC) is attracting increasing attention. The intestinal epithelial barrier separates luminal bacteria, metabolites and indigestible macromolecules from the subjacent lamina propria which contain leucocyte populations that contribute to tissue homeostasis in various contexts. Chemotherapy, radiation and pathogenic immune responses can lead to intestinal injury which is followed by tissue regeneration. However, exceeding damage can lead to impaired tissue repair and breakdown of the intestinal barrier function. This insufficient epithelial regeneration and barrier dysfunction subsequently results in disrupted intestinal immune homeostasis and is associated with multiple inflammatory disorders such as inflammatory bowel diseases, cancerogenesis or graft-versus-host disease (GVHD) after allogeneic hematopoietic stem cell transplantation (allo-HSCT).

The molecular pathways that regulate epithelial tissue repair during acute tissue damage are largely unexplored. Type I Interferons (IFN-I) are currently emerging as important modulators of intestinal homeostasis and evidence suggests that IFN-I fulfill protective roles in intestinal homeostasis suggesting that strategies to modulate innate immunity may be of therapeutic value for the treatment of intestinal inflammatory conditions. Along these lines, the IFN-I inducing cytosolic nucleic sensing RIG-I/MAVS pathways was shown to protect mice from chronic tissue damage such as experimental DSS-colitis, whereas the DNA sensing receptor AIM2, which is known to regulate DNA-induced inflammasome activation in murine myeloid cells, was discovered to promote irradiation induced tissue injury [1, 2]. Nonetheless, only little is known about the homeostatic functions of the RNA sensing RIG-I/MAVS or DNA- sensing cGAS/STING pathway during clinically relevant tissue damage such as irradiation, chemotherapy or immune-mediated intestinal tissue damage.

Our data suggest that activation of cytosolic nucleic sensing pathways including RIG-I/MAVS or cGAS/STING during allo-HSCT result in protective IFN-I signaling that maintain gut epithelial barrier integrity [3]. We used models of acute tissue damage including irradiation, chemotherapy or GVHD following allo-HSCT to examine epithelial barrier function and tissue repair mechanisms of IECs and the intestinal stem cell (ISC) compartment *in vivo* and *in vitro*. Mice lacking MAVS or RIG-I were more sensitive to irradiation- and chemotherapy-induced intestinal injury and developed worse GVHD compared to control mice. This observation was associated with reduced intestinal barrier integrity as evidenced by enhanced histopathology, permeability and neutrophil granulocytes infiltration. This data are consistent with a recent report of us showing that intestinal neutrophil granulocytes infiltration reflects intestinal epithelial barrier injury after acute tissue damage [4]. In contrast to RIG-I^−/−^ or MAVS^−/−^ mice, targeted activation of RIG-I by 5´-triphosphate RNA (3pRNA) augmented intestinal barrier function and regeneration. Importantly, RIG-I agonists had to applied during the onset or directly before induction of tissue damage, whereas later administration worsened the course of GVHD. Mechanistically, we found that correctly timed administration of RIG-I agonists enhanced growth of intestinal organoids which can be used as an *in vitro* model to study the regeneration capacities of the ISC compartment. Furthermore, RIG-I activation promoted the production of the antimicrobial peptide regenerating islet–derived protein 3 γ (RegIIIγ). In addition, we found that 3pRNA application induced IFN-I response in intestinal epithelial cells which mediated the observed regenerative and barrier function promoting effects. Notably, targeted activation of the cGAS/STING pathway also protected intestinal barrier function and reduced GVHD while STING-deficient mice developed more severe GVHD compared to control mice. Our study is in line with a recent report linking intestinal IFN-I signaling to enhanced intestinal epithelial turnover and repair and another report showing that type I IFN signaling protects from GVHD [5, 6].

Interestingly, a very recent study revealed that an intestinal microbiota derived metabolite (desaminotyrosine, DAT) protects from influenza infection via enhanced type I IFN signaling. Furthermore DAT-producing bacteria could rescue antibiotic-treated influenza-infected mice which usually show enhanced mortality [7]. Given that treatment of allo-HSCT recipients with broad-spectrum antibiotic is associated with the breakdown of intestinal homeostasis and increased mortality in human patients and mice [8], it is reasonable to speculate that the protective effects of the intestinal microbiota during GVHD may also be mediated via bacterial metabolites like DAT and / or endogenous nucleic acid ligands generated during genotoxic stress in order to amplify protective IFN-I signaling.

Although the direct target cells of these respective ligands including ISC or Paneth cells or intestinal immune cells and the intracellular signaling cascades triggering epithelial regeneration remain to be determined, our study paves the way to explore the benefits of RIG-I and STING activation in allo-HSCT recipients to circumvent or ameliorate GVHD development.
